# Dr. Pigot on Purging in Typhus

**Published:** 1812-06

**Authors:** J. M. B. Pigot

**Affiliations:** Memb. Royal Med. Society of Edinburgh, and Physician to the Chester General Infirmary. Nicholas-street, Chester


*To the Editors of the Medical and Physical Journal.*

GENTLEMEN,

SOME years have now elapsed since, by the publication of Dr. Hamilton's excellent Practical Treatise on Purgative Medicines, their utility in typhus fever was fully demonstrated. I am, however, led to suppose, that by the majority of practitioners these remedies are not sufficiently relied upon. I am persuaded the more the practice is made public, the more it will be followed. But, in order to render the exhibition of purgatives in typhus most beneficial, it is of the first consequence that they be regulated solely by the effect produced, and not by the number of grains administered. Many persons, from having imbibed an erroneous theory, dread the debilitating effects of purging in fever; others employ doses which irritate, without producing perfect evacuation, and of course exasperate all those symptoms they were intended to alleviate, and thus bring the practice into disrepute through the timidity of the practitioner. I have now such perfect confidence in purgatives, that in the incipient stages of typhus I have no fear of trusting to them entirely; and my journal of cases under my care, in the fever-wards of the General Infirmary of this city, will amply testify the happy result. It is necessary, however, that they be confined to some plain rules of practice, which a little attention will render sufficiently evident. By copying a case or two from my prescription-book, and adding a short comment, I trust I shall not be occupying an altogether useless space in your widely-circulated Journal.

Thomas Martin, æt. 24, admitted Dec. 26th. Has great determination of blood to the head, with delirium. Skin dry. Thirst urgent. Heat 100. Bowels bound. Pulse 104. Has been ill six days, and was taken with vomiting. Was brought in a cart from Belgrave to-day.* The nurse has found it necessary to use the strait waistcoat.

* About three miles from Chester.

Statim

Statim capiat Calomelanos gr. x. cu. ℥j. Mist. Salin: Aper.*

Repet. Mist. quâq. horâ.

Vesicatoria temporibus et Haust. Anod. Diaph.† vespere.

Dec. 27th. Has had *upwards of twenty stools.* Head much relieved. Delirium gone. Waistcoat removed. Heat less, and pulse more soft. Urine plentiful.

Cont. Mist. Aper. quâq. horâ.

Repet. Calomel gr. x. vesp. et Haust. Anod. Diaph.

28th. Twenty stools since last visit. Pulse 100, and soft. Skin moist. Makes much urine. Eat some dinner with appetite. No pain of head. Some pain of breast, with cough.

Mist. et Haust. ut antea. Vesp. Cal. gr. v.

29th. Twelve stools since last visit. Makes much urine. Pulse 82, and soft. Heat natural. Complains of cough, and pain of chest, with some expectoration.

Omitt. Mist. Aper. Cont. Haust. Appl. Vesicatorium sterno.

℞. G. Myrrhæ G. Ammon. aa ʒſs. ter s. a. c.

Aq. Ammon. Acet. ℥j. Decoct. Cinchonæ ℥vij. M. sumat ℥j. c. ʒſs. pulveris Cinchonæ quâq. horâ.

Habt. Vin. Rubri Lusitanici ℔ſs. c. triplice Aquæ.

30th. Seven stools since last visit. Pulse 80, and natural. Skin rather dry. Much urine. Sleeps badly.

Fiat Haust. c. gtt. xxxv. T. Opii. Calomelanos gr. v. alia ut hire.

31st. Four stools. Blister rose well. Cough and breathing relieved.

Vesp. Cal. gr. v.

Jan. 1st. Four stools. Pulse 80. Cough almost gone. Gains strength fast.

Medicam. ut antea. Convalescent.

No further report was taken, and the man left the hospital perfectly well on the 11th.

The above case, though by no means so copiously stated as it would have been, had any idea of publication existed at the time of recording it, amply shews how quickly the worst symptoms of fever may be removed by the vigorous administration of purgatives. In my opinion, in a much shorter space of time than by any other mode of practice which has hitherto been adopted. Until the pulse be re-

---

* ℞. Magnes. Sulpt. ℥j. Mann. ℥ſs. Solve ex Aq. Menth. ℔ſs. f. Mist.

† ℞. Aq. Ammon. Acet. ʒij. T. Opii gr. xxv. Aq. Menth. ℥j. Mf. Haustus.

 duced duced to its natural standard, we need be under no fear of continuing the intestinal evacuation; though at the same time it may be necessary to support the vis vitæ by proper cordials.

When the pulse has been reduced and becomes soft, it will usually happen that all the other symptoms subside. The strength of the patient must not then be unnecessarily exhausted by a longer continuance of purging, though it is essentially necessary that the bowels be kept open. Bark, wine, and nourishing diet, will in a few days restore the strength in a wonderful manner.

I shall take the liberty of subjoining another case, of a female, where the cure was not so quickly effected, owing to the unusual torpidity of the bowels.

Sarah Hopley, æt. 22, brought into the female fever-ward Jan. 7th, with great pain of head and back. Skin hot and dry. Thirst urgent. Tongue furred. Pulse 106, and full. Was taken ill on Friday (3d). Had three stools yesterday. Taken some medicines. Does not know what.

℞. Calomelanos et Zinziberis aa gr. v. Pulv. Jalap ʒss. Mf.
Pulvis statim sumendus.
Sumat. Mist. Sal. Aper. ℥j. quâq. semihorâ.

8th. Pain of head not so severe, but complains of her throat, which is somewhat swelled. Has had only one stool, having taken her mixture only every hour. Pulse 114, and full. Skin hot. Cheeks flushed.

Mixture every *half* hour.
Vesicatoria pone aures.

9th. Blisters rose, and discharged well. Head and throat much relieved. Has had only seven or eight stools. Pulse 116. Skin still hot, and cheeks flushed.

Statim capiat pulv. sequent.

℞. Calomelanos gr. x.

Pulv. Jalap ʒss.

—— Zinziberis gr. v. Mf. pul.

Mist. ut antea.

10th. Has had only ten stools. Pulse 116. Pain of head gone. Thirst less. Skin moist. Has a troublesome cough, and some pain of chest.

Statim sterno admoveat. Vesicatorium.

Fiat Mist. c. ℥ss. Tinc. Jalapæ et sumat ℥ij. quâq. semihorâ.

11th. About fifteen stools, all very fluid, and of natural colour. Pulse still 108, but much softer. Blister rose well. Cough much better. No pain.

Sumat Mist. ut heri.

12th.

12th. About thirteen stools. Pulse 108, and full. No pain or uneasiness of any sort. Has eaten some rice-pudding.

Sumat Mist. ut antea et vespere Haust. seq.

℞. T. Digitalis gtt. xv.

Aq. Ammon. acet. ℥ſs.

Aq. Menth. ſs. ℥j. Mf. Haust.

13th. About twelve stools. Draught did not cause any sickness. Slept well. Pulse 92. Complains only of weakness of her legs.

Cont. Mist. u. a. et Haust. vesp.

14th. About the same number of stools as yesterday. Pulse 82. Had meat and potatoes yesterday, which agreed well.

Omitt. Mist. Aper. et Haust. Digitalis.
Porter ℔j.

℞. Decoct. Cinchonæ ℥viij. T. Columbæ ℥ſs. M. capt ℥j. ter die.

15th. Four stools. Pulse 86. Mixture and porter as yesterday.

Vini Domestici* ℔ſs. Convalescent.

No further report. Went out perfectly well in a few days.

In taking a review of this case, we observe the medicines on the 7th failed in their operation, and the pulse was materially accelerated on the 8th. On the 9th, not more than *half* the evacuation, which I expected had been procured, and the pulse rose to 116. Notwithstanding the augmented doses, no diminution of arterial energy was perceptible till the 11th, when about thirty-three intestinal evacuations had been procured. The pulse then only fell from 116 to 108. I well knew all the unpleasant symptoms would return, if the same vigorous plan was not pursued till the arterial energy was more exhausted. This was not effected till the 13th, when twenty-five more stools had been procured, and the pulse sunk to 92, in which reduction I attribute something to the Digitalis. The violence of the disease was subdued from this time, and on the 14th, when the pulse was brought down to 82, I thought it safe to throw aside the purging and adopt the tonic plan, which succeeded to my wishes.

The above cases I might have made more copious from my perfect recollection of them; but I have not thought myself at liberty to make any alteration in reports publicly taken in the presence of nurses, apprentices, and pupils, attending the practice of the hospital, who have daily an opportunity of comparing the record with the state of the patient.

* A good-bodied raisin wine.

The exhibition of calomel in the large doses of the above, and a great number of similar cases, I have seldom found to affect the salivary glands. When, however, that has been the case, the recovery has been uniform and speedy. Many physicians are of opinion, that in the continued fevers of our island there is a certain determined duration, a stated course for them to run, which, though the powers of medicine may alleviate urgent symptoms, cannot cut short the disease, unless very soon after its commencement. In this opinion, so paralysing to our exertions, so humiliating to our art, I cannot coincide. Even those who favor the above opinion, differ among themselves concerning the length of time within which, from its commencement, they may be able to cut short the disease. The longer the assistance of the physician is delayed, of course the more difficult will be the cure, and the probability of recovery less. We are not, however, to despair of effecting a cure by purgative medicines, even in the more advanced stages of the disease. So long as the pulse remains above the strength and standard of health, we may employ the vigorous means above recommended, with the utmost confidence of a happy termination. It would be no difficult matter to shew, from the oldest writers downwards, that, even in the last stage of typhus, nature has suddenly relieved the patient by large intestinal evacuations, and thence called critical. I shall forbear, however, from extending these observations, and rejoice if I induce the medical men, in different parts of Great Britain, to give the result of their practice, in regard to purging in fever; so that, if good, its utility may be established, and circulated as widely as possible.

I have the honor to be, Gentlemen,

Your most obedient servant,

J. M. B. PIGOT, M.D.

Memb. Royal Med. Society of Edinburgh, and Physician to the Chester General Infirmary.

*Nicholas-street, Chester, April* 10, 1812.

